# Maternal proximity to Central Appalachia surface mining and birth outcomes

**DOI:** 10.1097/EE9.0000000000000128

**Published:** 2021-01-25

**Authors:** Lauren G. Buttling, Molly X. McKnight, Korine N. Kolivras, Shyam Ranganathan, Julia M. Gohlke

**Affiliations:** a Department of Population Health Sciences, Virginia Tech, Blacksburg, Virginia; b Department of Geography, Virginia Tech, Blacksburg, Virginia; c Department of Statistics, Virginia Tech, Blacksburg, Virginia.

**Keywords:** Appalachia, Birth outcomes, Surface mining, Preterm birth, Low birth weight, Birthweight, Coal

## Abstract

Maternal residency in Central Appalachia counties with coal production has been previously associated with increased rates of low birth weight (LBW). To refine the relationship between surface mining and birth outcomes, this study employs finer spatiotemporal estimates of exposure.

**Methods:**

We developed characterizations of annual surface mining boundaries in Central Appalachia between 1986 and 2015 using Landsat data. Maternal address on birth records was geocoded and assigned amount of surface mining within a 5 km radius of residence (street-level). Births were also assigned the amount of surface mining within residential ZIP code tabulation area (ZCTA). Associations between exposure to active mining during gestation year and birth weight, LBW, preterm birth (PTB), and term low birth weight (tLBW) were determined, adjusting for outcome rates before active mining and available covariates.

**Results:**

The percent of land actively mined within a 5 km buffer of residence (or ZCTA) was negatively associated with birth weight (5 km: β = –14.07 g; 95% confidence interval [CI] = –19.35, –8.79, *P* = 1.79 × 10^–7^; ZCTA: β = –9.93 g; 95% CI = –12.54, –7.33, *P* = 7.94 × 10^–14^). We also found positive associations between PTB and active mining within 5 km (odds ratio [OR] = 1.06; 95% CI = 1.03, 1.09, *P* = 1.43 × 10^–4^) and within ZCTA (OR = 1.04; 95% CI = 1.03, 1.06, *P* = 9.21 × 10^–8^). Positive relationships were also found between amount of active mining within 5 km or ZIP code of residence and LBW and tLBW outcomes.

**Conclusions:**

Maternal residency near active surface mining during gestation may increase risk of PTB and LBW.

## Abstract 2

Supplemental Digital Content is available in the text.

**What this study adds**

Our analysis improves upon previous studies that suggest low birthweight is heightened in Appalachia counties with mining by employing fine spatial resolution satellite images of surface mines between 1987 and 2015 and address-level birth records. By determining the mining area within a 5 km buffer of the maternal residence address during the gestation year, we can employ a modified difference in difference approach, adjusting for baseline differences in outcomes between births occurring in locations that did not experience any mining across the period of study versus locations that experienced mining.

## Introduction

Since 1990, surface mining, typically through mountaintop removal or contour mining, has made up over half of Appalachian mining activities.^1,2^ This method allows for nearly total recovery of coal and requires a smaller workforce than underground mining.^3^ However, the rock and earth waste associated with surface mining can be equal to or greater than the amount of coal produced.^4^ Previous research has established a relationship between the inhalation of particulate matter (PM) and adverse birth outcomes.^5–9^ A few studies have provided evidence of increased PM exposure and related negative health outcomes near Central Appalachia surface mining sites.^6,10^

PM from surface mining includes particulates that can be small enough to reach the fetal bloodstream and trigger maternal inflammatory pathways.^6,11^ Specifically relevant to coal truck emissions, increased exposure to traffic-related PM during gestation has been associated with an increased risk of low birth weight (LBW, <2,500 g), preterm birth (PTB, birth at <37 weeks gestation), and term low birth weight (tLBW, born at ≥37 weeks gestation and weighing <2,500 g).^12–15^ Increased proximity to major roadways has also been associated with an increased risk of LBW, PTB, and tLBW.^9,16,17^ Another study concluded that risk of early pregnancy loss increases in female mice with increasing exposure concentrations of diesel particulate emissions.^18^ Diesel emissions are expected to be greater around active surface mines, with a one study of an active surface mine in Appalachia cataloging 14 diesel haul trucks each averaged 8.68 working hours per day over a year.^19^

Previous studies have demonstrated increased adverse birth outcomes in Appalachian coalfield areas compared with other Appalachian areas at a relatively coarse county-level scale, comparing coal producing to noncoal producing counties.^20,21^ Through classifying Landsat satellite imagery (30 m resolution) and geocoding individual birth records, this study determines the association between birth outcomes and surface mining by developing a fine-scale spatiotemporal characterization of historical surface mining sites from 1986 to 2015 to determine proximity to active surface mining during gestation using maternal residential addresses, mailing address, or ZIP code from 1990 to 2015. We hypothesize that increased exposure to active mining, when compared with areas before active mining, is associated with an increase in the odds of PTB, LBW, tLBW, and decreased birth weight at both street and ZIP code-level spatial scales.

## Methods

### Birth records

A uniform dataset was created from 409,394 birth records (Supplemental Figure 1; http://links.lww.com/EE/A116), which were provided by Tennessee (TN), Virginia (VA), West Virginia (WV), and Kentucky (KY) health departments, and geocoded to the Central Appalachia counties as defined by the Appalachian Regional Commission.^22^ Formatting of birth records varied by state and changed over the time period of study, requiring several data processing steps. For the street-level analysis, maternal residence recorded at birth were geocoded to street address using residential or mailing address and parsed to remove records with only Post Office (P.O.) boxes. The street-level dataset is comprised of 194,084 records with mother’s geocoded street address, outcome variables, and covariates, described in more detail below.

Due to substantial missingness in the street-level dataset, a ZIP (United States Postal Service 'Zone Improvement Plan' area) code-level analysis was also conducted to preserve a greater sample size of 364,981 records, which uses maternal ZIP code to assign exposure. While the spatial resolution is reduced in this analysis compared with the street-level, it provides a finer scale analysis than existing studies. The ZIP code-level dataset was determined by including all street-level birth records with a valid ZIP code and all those where the respective ZIP code tabulation area (ZCTA) boundary intersects with at least 2% of a county classified as within Central Appalachia by the Appalachian Regional Commission.^22^ This accounts for ZCTAs that cross county lines and where ZCTA centroids may be outside of a Central Appalachia county. All 364,981 birth records with valid ZIP codes geolocated using the postal locator created from Esri’s 2013 StreetMap dataset and no missing covariates were included in the ZIP code-level analysis (Supplemental Figure 1; http://links.lww.com/EE/A116). We used ZIP codes on the vital records to geographically match birth records with ZCTA boundaries. Although we recognize the algorithms used to create these boundary files vary,^23^ we refer to ZCTAs as ZIP codes in the rest of the manuscript for the sake of clarity.

This study was reviewed and approved by the Virginia Tech Institutional Review Board (IRB) (No. 16-898), Virginia Department of Health IRB (No. 40221), West Virginia Department of Health and Human Resources, Kentucky Cabinet for Health and Family Services IRB (No. FY17-23), and Tennessee Department of Health IRB (No. 972154). Tennessee Department of Health data used in this study were obtained from the vital statistics program, Tennessee Department of Health (TDH). Use of these data does not imply TDH agrees or disagrees with any presentations, analyses, interpretations, or conclusions herein.

### Delineation of surface mine extents

Active surface mines within Central Appalachia surface mining permit boundaries were determined for each year between 1986 and 2015 using Landsat imagery characterization, as described in detail in Marston and Kolivras.^24^ Briefly, evidence of mining activity was determined by transitions from vegetative land cover to bare ground within surface mining permit boundaries, while considering seasonality, and build from previous methods for automated methods for determining surface mine extents from satellite-derived datasets. Marston and Kolivras^24^ present comparisons with previously published methods for determining surface mining extents using remotely sensed data,^25–28^ and spot checking of satellite-derived estimates with aerial photography, suggesting general consistency across methods. For the purposes of this study, all active mine boundaries were filtered for continuous polygons of 40 acres or more, based on the land area required for economic viability of a surface mine.^29^ For each year of analysis, mined land was categorized into (1) pre-mining areas, which was defined as land that was not experiencing active mining during that year but will have active mining in later years; (2) actively mined area; and (3) post-mining areas, which was classified as land actively mined in prior years of analysis, but no evidence of active mining during the exposure year.

### Exposure variables

For the street-level analysis, the amount of pre, active, and post-mining land area within a 5 km, circular buffer of mother’s address was quantified using ArcGIS Pro (Esri Inc., Redlands, CA). Note that amount of mining is measured as land area disturbed, as described above, and does not refer to the tonnage of coal mined. The exposure year for a given birth was determined to be the year of the majority of gestation (≥50% of gestation), 1989 to 2015. Birth records missing gestation length (0.1%) were removed from analysis, as their exposure year could not be determined. Maternal residence location was matched to the respective surface mining polygons for the majority gestation year to determine the area of each buffer’s intersection with surface mine category boundaries (Supplemental Figure 2; http://links.lww.com/EE/A116). These intersections were calculated in square meters and percent area of the total buffer. All birth records that did not have any measurable polygon intersection were given a value of 0 for the generated exposure metrics. A similar methodology was used for the ZIP code-level analyses, with calculation of the percent of land defined as pre, active, and post-mining within the ZCTA boundaries of mothers’ residential or mailing address during the majority year of gestation.

### Covariates and outcome variables

Demographic variables included in birth records were considered covariates in statistical analyses. Mother’s age was included and categorized into groups of those under 18, 18–35, and over 35 years for analysis since previous studies have shown increased risk of adverse birth outcomes at younger and older maternal ages.^30^ Parity was determined from the child’s birth order (Virginia, West Virginia, and Tennessee births) or by number of previous children living (Kentucky births) and categorized into 1, 2, 3, and 4 or more births, based on studies that have shown an association between number of previous births and risk of adverse birth outcomes.^31,32^ Mother’s education was classified as 8th grade or less, 9th to 12th grade, or any post high school education with or without a degree. Payment method for delivery costs (Medicaid, private insurance, self-pay, or another form of payment) was included as an indicator of socioeconomic status.^33,34^ Kentucky records from 1990 to 2013 did not include any information on payment and those from West Virginia only determined if Medicaid was used from 1990 to 2013, leaving only 99,595 street-level records and 173,232 ZIP code-level records for analysis when these covariates are added. Race reported on the birth record was categorized as White, Black or African American, or other based on the maternal race field from KY, WV, and TN birth records and child’s race field for VA records.^35^ Mother of Hispanic origin was recorded with exception of West Virginia records from 1990 to 2013. Child’s sex and any tobacco use during pregnancy were classified from birth records. Eleven percent of records were removed from the street and 9% from the ZIP code-level datasets after removing missingness in all covariates other than payment method and Hispanic origin (Supplemental Figure 1; http://links.lww.com/EE/A116).

This study only included singleton births due to the differing rates of PTB and LBW in plural births.^36^ Gestation lengths of 21 to 45 weeks and birth weights of 200 g or greater were considered valid for our study sample.^37,38^ Gestation length and birth weight were used to determine the outcome variables, PTB, LBW, and tLBW.

### Study design

A variant of the generalized difference in difference or controlled interrupted time series approach is employed,^39,40^ with amount of pre-mining within the 5 km buffer (or ZCTA boundary) serving as the pretreatment condition within areas that will subsequently be mined, and amount of active mining serving as the treatment measure. Areas with no mining throughout the time period are considered untreated controls and secular trends in birth outcomes over the study period are included. Because pre and active mining areas occur simultaneously within 5 km buffers (or ZCTAs), regression models with the percent of land categorized as pre-mining as the exposure variable is evaluated initially. Subsequent models adding the percent of land categorized as actively mined (primary exposure variable of interest) also evaluate the joint effects by inclusion of an interaction term between pre and active mining. Finally, to account for potential lingering effects after active mining operations are completed, percent of area that was previously mined (post-mining areas) within the 5 km buffer around the maternal residence (or ZCTA) were included in a third set of regression models, with interaction terms between pre and active, and active and post mining to account for joint effects. Since post-mining areas are typically next to active mining areas in the current year, the amount of each within a 5 km buffer are correlated (*R*^2^: 28.9); therefore, collinearity may make it difficult to tease apart effects of active mining versus effects from previous mining that occurred in the area. To illustrate the progression from pre to active, to post-mining, Supplemental Figure 3; http://links.lww.com/EE/A116 shows the amount of pre, active and post-mining, as a percent of total area, in four representative ZCTAs with a relatively high amount of mining (>30% mined), over the study period.

### Statistical analyses

Birth weight, PTB, LBW, or tLBW were dependent variables in separate logistic regression models using the street or ZIP code-level exposure estimates following the general equation below.


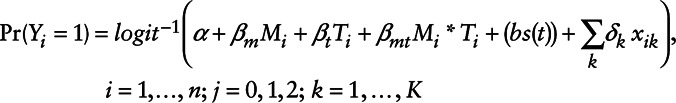


where *Y_i_* is the dichotomous birth outcome being modeled; *M_i_* is the % of the land area within the 5 km buffer around the maternal residence that is categorized as pre-mining area for individual *i*; 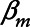 is the change odds of the birth outcome per % increase in pre-surface mining area within the 5 km buffer; *T_j_* is the % of the 5 km buffer around the maternal residence that is categorized as active mining for individual *i*, with 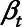 representing the change in odds of the birth outcome per % increase in active mining within the 5 km buffer and 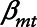 representing the joint effect on the outcome when both pre and active mining are present; 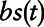 in years represents a spline with 4 degrees of freedom (bs() function in the R spline package) to adjust for secular trends in outcomes; and 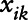 are the *K* covariates for individual *i* described further below.

Models with pre mining only, pre and active mining (model represented above), and pre, active and post mining, including interaction terms, were evaluated. All models include the categorical covariates of mother’s age, mother’s race, mother’s reported tobacco use during pregnancy, mother’s education, previous births, birth record state (KY, VA, TN, or WV), and child’s sex. Payment method was omitted from the primary analysis as its inclusion removed 48.7% and 52.5% of records from the street and ZIP code-level analysis, respectively, due to missingness of these fields in KY and WV records for several of the years within the period of study. Mother’s Hispanic origin was not used in the primary analysis as it removed 6.3% and 3.4% of records from the street and ZIP code-level analysis, respectively. Both of these covariates were included in a secondary analysis, and inclusion of birth record state in all models additionally adjusts for missingness associated with birth records received from a given state. Linear (birthweight) and logistic (PTB, LBW, and tLBW) models were run in R using the glm() function. Results are expressed as the change in birthweight (grams) and 95% confidence interval (CI) (or odds ratio [OR] and 95% CI for a PTB, LBW, or tLBW) per 1% increase of a boundary’s land occupied by mining activity category. Akaike Information Criteria output is used to compare models with pre only, pre and active, or pre, active and post categories of mined land.

## Results

### Street-level population characteristics

**Figure 1.**
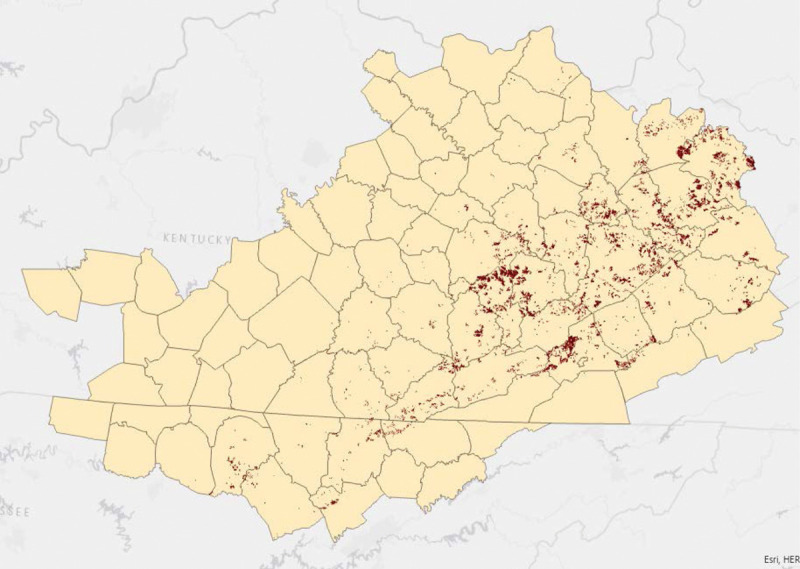
Cumulative active surface mining area from 1989 to 2015 in Central Appalachia counties.

Of the residential addresses able to be geocoded to the street-level, mothers were primarily between 18 and 35 years of age (90%), mostly White (97%), with some high school education (56% had a highest education of 9–12th grade), and mostly did not report smoking during pregnancy (70%). Twelve percent of births were exposed to active mining within 5 km of maternal residence during gestation (Table 1). All area defined as being actively surface mined during at least 1 year within the time period of study is mapped in Figure 1.

**Table 1.** Characteristics of singleton births unexposed and exposed to active mining included in the street and ZIP code-level analyses

Characteristic	Street-level		ZIP code-level	
	No mining within 5 km, n = 170,351, n (%)	Active mining within 5 km, n = 23,733, n (%)	No mining in ZCTA boundary, n = 261,608, n (%)	Active mining ZCTA boundary, n = 103,373, n (%)
Mother’s state of residence				
Kentucky	149,652 (87.8)	19,711 (83.1)	230,610 (88.2)	88,652 (85.8)
Tennessee	10,215 (6.0)	1,279 (5.4)	9,265 (3.5)	2,269 (2.2)
Virginia	3,195 (1.9)	1,533 (6.5)	13,923 (5.3)	9,290 (9.0)
West Virginia	7,289 (4.3)	1,210 (5.1)	7,792 (3.0)	3,180 (3.1)
Child’s sex				
Male	87,776 (51.5)	12,279 (51.7)	134,632 (51.5)	53,298 (51.6)
Female	82,575 (48.5)	11,454 (48.3)	126,958 (48.5)	50,093 (48.4)
Mother’s race				
White	165,163 (97.0)	23,258 (98.0)	252,692 (96.6)	100,331 (97.0)
Black	2,747 (1.6)	275 (1.2)	3,349 (1.3)	814 (0.8)
Other	2,441 (1.4)	200 (0.8)	5,549 (2.1)	2,246 (2.2)
Mother’s age (yr)				
18–35	153,784 (90.3)	21,566 (90.9)	235,196 (89.9)	93,105 (90.1)
<18	7,765 (4.6)	1,058 (4.5)	13,907 (5.3)	5,747 (5.6)
>35	8,802 (5.2)	1,109 (4.7)	12,487 (4.8)	4,539 (4.4)
Previous births				
0	74,310 (43.6)	10,403 (43.8)	113,921 (43.6)	45,424 (43.9)
1	57,750 (33.9)	8,202 (34.6)	88,245 (33.7)	35,201 (34.1)
2	25,552 (15.0)	3,567 (15.0)	39,146 (15.0)	15,281 (14.8)
3	8,192 (4.8)	1,066 (4.5)	12,787 (4.9)	4,990 (4.8)
4 or more	4,547 (2.7)	495 (2.1)	7,491 (2.9)	2,495 (2.4)
Mother’s education (yr)				
<9	6,458 (3.8)	607 (2.6)	13,138 (5.0)	4,663 (4.5)
9–12	95,633 (56.1)	13,729 (57.8)	156,211 (59.7)	63,554 (61.5)
>12	68,260 (40.1)	9,397 (39.6)	92,241 (35.3)	35,174 (34.0)
Reported tobacco use during pregnancy				
No	119,868 (70.4)	15,962 (67.3)	179,547 (68.6)	68,303 (66.1)
Yes	50,483 (29.6)	7,771 (32.7)	82,043 (31.4)	35,088 (33.9)
Payment				
Medicaid	53,957 (31.7)	9,320 (39.3)	77,278 (29.5)	34,967 (33.8)
Private insurance	27,801 (16.3)	4,213 (17.8)	38,952 (14.9)	14,747 (14.3)
Self-pay	2,729 (1.6)	195 (0.8)	4,051 (1.5)	786 (0.8)
Other	1,182 (0.7)	198 (0.8)	1,734 (0.7)	713 (0.7)
NA	84,682 (49.7)	9,807 (41.3)	139,575 (53.3)	52,178 (50.5)
Mother’s Hispanic origin				
Not Hispanic	157,789 (92.6)	22,116 (93.2)	250,195 (95.6)	99,506 (96.2)
Hispanic	1,913 (1.1)	100 (0.4)	2,502 (1.0)	482 (0.5)
NA	10,649 (6.3)	1,517 (6.4)	8,893 (3.4)	3,403 (3.3)

NA indicates not available.

**Figure 2.**
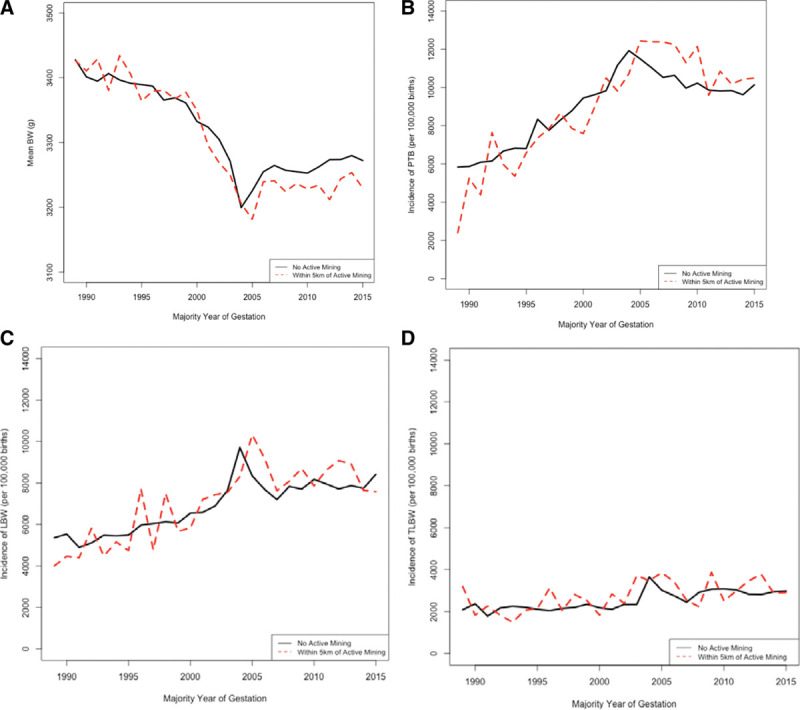
Trends in birth outcomes over study period. A, Average birthweight over time for residence within 5 km of active mining versus not within 5 km of active mining in Central Appalachia from 1989 to 2015. B, Incidence rate of preterm birth (per 100,000 births) within 5 km of active mining and those unexposed from 1989 to 2015. C, Incidence rate of low birth weight births (per 100,000 births) within 5 km of active mining and those unexposed from 1989 to 2015. D. Incidence rate of term low birth weight births (per 100,000 births) within 5 km of active mining and those unexposed from 1989 to 2015. BW indicates birthweight.

Graphical examination of the incidence rate of adverse birth outcomes over the study period in both births unexposed and exposed to active mining within 5 km of maternal residence suggest generally similar trends through 2005 (Figure 2). Birth weight and PTB rates appeared to be minimally different between the two groups until 2005, where births in mining areas exhibited a consistently lower average birth weight and higher rates in PTB. Overall trends are consistent with national trends from 1990 to 2013, when induced labors and cesarean deliveries increased in popularity throughout the United States and births became much more likely to occur during weeks 37–39 than past 40 weeks.^41^ Results from this previous study suggest that the average US birthweight would have increased over this time period, if rates of induced labors and cesarean deliveries remained unchanged. The rate of PTB in Central Appalachia also followed national trends, with an observed nationwide increase in PTB from 1998 to 2006, peaking in 2006, and generally decreasing between 2006 and 2015.^42^

### Street-level model results

The percent of pre-mining area within the 5 km buffer around the maternal residence is not associated with birth outcomes (Supplemental Table 1; http://links.lww.com/EE/A116), suggesting areas that will be mined later in the study time period have similar birthweights, and rates of PTB, LBW, and tLBW as locations without mining throughout the study period. For births that occurred within 5 km of active mining, the percent of a 5 km buffer occupied by active mining ranged from <0.001% to 15.76%. In adjusted linear regression analyses, for every 1% increase of active mining within a 5 km buffer of maternal residence, a 14 g decrease in birth weight is estimated (β = –14.07 g; 95% CI = –19.35, –8.79, *P* = 1.79 × 10^–7^). Using these regression results with a maximum observed exposure of 15.76% within a 5 km buffer of residence, active mining is associated with a decrease in birth weight of up to 222 g.

In the adjusted logistic regression analyses, every 1% increase in active mining within 5 km of maternal residence was associated with a 6% increase in the odds of a PTB (OR = 1.06; 95% CI = 1.03, 1.09, *P* = 1.43 × 10^–4^) and LBW (OR = 1.06; 95% CI = 1.02, 1.09, *P* = 1.37 × 10^–3^) (Table 2). The estimated odds of PTB and LBW were 2.5 times higher for residences with the highest (15.76%) active mining within 5 km buffers. Every 1% increase in mining within 5 km of maternal residence was associated with a 2% increase for tLBW (OR = 1.02; 95% CI = 0.97, 1.08, *P* = 0.440) (Table 2).

**Table 2.** Odds ratios (95% CIs) for associations between adverse birth outcomes and amount of active mining within 5 km of maternal residence

Outcome variable	Active mining within 5 km of maternal residence (per 1% increase)	
	OR (95% CI)	*P*
Preterm	1.06 (1.03, 1.09)	1.43 × 10^–4^
Low birth weight	1.06 (1.02, 1.09)	1.37 × 10^–3^
Term low birth weight	1.02 (0.97, 1.08)	0.440

Adjusted for mother’s age, mother’s race, mother’s tobacco use during pregnancy, mother’s education, parity, year of majority of gestation, state, and child’s sex.

Post-mining area ranged from <0.001% to 41.74% for those addresses with any mining within the 5 km buffer. A 1% increase in post-mining activities within the 5 km buffer was associated with a 2.10 g decrease in birth weight (β = –2.10 g; 95% CI = –3.59, –0.61, *P* = 5.80 × 10^–3^), of up to 87.5 g at locations with the highest post-mining. PTB, LBW, and tLBW were also analyzed and showed similar relationships between exposure to post-mining areas and adverse birth outcomes (Supplemental Table 2; http://links.lww.com/EE/A116).

Models with pre-mining that limits records for analysis but allows for inclusion of Hispanic origin of mother and payment method as covariates are similar to model results described above (Supplemental Table 3; http://links.lww.com/EE/A116). In models including payment method and Hispanic origin of mother, a negative association between amount of active surface mining within 5 km and birth weight (β = –15.73 g; 95% CI = –22.16, –9.29, *P* = 1.69 × 10^–6^) was found. Effect estimates for PTB, LBW, and tLBW were similar to models described above (Supplemental Table 4; http://links.lww.com/EE/A116). However, because these models remove all birth records received from WV between 1990 and 2009 and those from KY between 1990 and 2013, inclusion of these variables reduced our sample size by 56.4% (Table 1).

### ZIP code-level population characteristics

The ZIP code-level analysis allowed for the inclusion of an additional 170,897 records of Central Appalachia residents that could not be included in the street-level analysis. ZIP code-level exposures allow us to capture outcomes of mothers who reported a P.O. box address, did not have a complete address recorded, or were unable to be geocoded to street address. The birth records able to be geocoded to Central Appalachia’s ZCTA boundaries were demographically comparable to the street-level dataset, with mothers largely between 18 and 35 years of age (90%), and mostly White (97%), with some high school education (60% had a highest education of 9–12th grade), and the majority did not report tobacco use during pregnancy (68%). Twenty-eight percent of births had reported maternal residences in ZCTAs with active mining activity during the year in which the majority of gestation occurred (Table 1).

### ZIP code-level model results

Consistent with street-level analyses, the percent of pre-mining area within the 5 km buffer around the maternal residence was not associated with birthweight, PTB, LBW, or tLBW (Supplemental Table 5; http://links.lww.com/EE/A116).

For those births with active mining within maternal ZCTA during gestation year, the percent of the respective ZCTA boundary occupied by active mining ranged from <0.001% to 19.72%. A 10 g decrease in birth weight for every 1% increase of mining area within the maternal ZCTA boundary was estimated (β = –9.93; 95% CI = –12.54, –7.33, *P* = 7.94 × 10^–14^), suggesting an average decrease in birth weight of 196 g in the ZCTA year with the maximum observed % mining.

In the adjusted logistic regression analyses, every 1% increase in mining within the ZCTA was associated with a 4% increase in the odds of PTB (OR = 1.04; 95% CI = 1.03, 1.06, *P* = 9.21 × 10^–8^). The estimated odds of a PTB is 2.25 times higher in the ZCTA year with the most active mining. Every 1% increase in active mining was associated with a 3% increase in the odds of a birth being classified as LBW (OR = 1.03; 95% CI = 1.02, 1.05, *P* = 9.05 × 10^–5^) or tLBW (OR = 1.03; 95% CI = 1.00, 1.05, *P* = 0.046) (Table 3). Active mining is associated with a 70%–90% increase in the odds of LBW and tLBW, respectively, in the ZCTA year with the highest percent of active mining.

**Table 3.** Odds ratios (95% CIs) for associations between adverse birth outcomes and amount of active mining within maternal ZCTA in adjusted logistic model

Outcome variable	Active mining within ZCTA boundary (per 1% increase)	
	OR (95% CI)	*P*
Preterm	1.04 (1.03, 1.06)	9.21 × 10^–8^
Low birth weight	1.03 (1.02, 1.05)	9.05 × 10^–5^
Term low birth weight	1.03 (1.00, 1.05)	0.046

This regression was adjusted for mother’s age, mother’s race, mother’s tobacco use during pregnancy, mother’s education, parity, year of majority of gestation, state, and child’s sex.

The amount of land within maternal ZCTA that was classified as post-mining was also determined. For those births with any post-mining in their respective ZCTA, the percentage of land occupied by post-mining ranged from <0.001% to 40.59%. A 1% increase in post-mining activities within the 5 km buffer was associated with a 3.56 g decrease in birth weight (β = –3.56; 95% CI = –4.42, –2.70, *P* = 4.63 × 10^–16^). The relationship between PTB, LBW, and tLBW and exposure to post-mining areas within maternal ZCTA was similar to that of the street-level analysis (Supplemental Table 6; http://links.lww.com/EE/A116).

In sensitivity models including payment method and Hispanic origin of mother, results demonstrated no association with pre-mining (Supplemental Table 7; http://links.lww.com/EE/A116). There was a negative association between amount of active mining within maternal ZCTA boundaries and birth weight (β = –15.97 g; 95% CI = –19.60, –12.33, *P* =7.75 × 10^–18^) and positive associations with PTB, LBW, and tLBW (Supplemental Table 8; http://links.lww.com/EE/A116). Because these models removed all birth records received from WV between 1990 and 2009 and those from KY between 1990 and 2013, inclusion of payment method and maternal Hispanic origin in regression models reduced our sample size by 49.0% (Table 1).

Overall, error is minimized, as measured by Akaike Information Criteria, in models including active and post-mining for birthweight and PTB, whereas inclusion of post-mining does not improve the statistical models of LBW and tLBW (Supplemental Table 9; http://links.lww.com/EE/A116).

## Discussion

This analysis showed that maternal residence in close proximity to active surface mining at both spatial scales was associated with higher odds of adverse birth outcomes and demonstrated a negative relationship between birth weight and proximity to active mining after controlling for individual-level covariates available across birth records and potential differences in outcomes before active mining. Previous studies have associated adverse health and birth outcomes with proximity to surface mining activities and associated particulate matter crustal compounds.^5,10,20,21,43–45^ This is the first study to apply a quantification of active surface mines at a fine spatiotemporal resolution at address and ZIP code-levels.

Our findings add to the understanding of the effect of living near surface mining on PTB, LBW, tLBW, and birth weight. A recent study estimated the change in odds of LBW associated with the amount of coal produced in West Virginia counties from 2005 and 2007 through use of individual birth records.^21^ All West Virginia counties were included, which include areas outside of Central Appalachia. This study suggested that residence in a county with high amounts of coal production was associated with 16% increased odds of LBW (95% CI = 8%, 25%) after adjustment for available risk factors. The present study differs from this previous study in several ways, including differences in the covariates available from the West Virginia Birthscore Dataset, such as mother’s marriage status, alcohol consumption, and prenatal care, the inclusion of non-Central Appalachia mining activity, coal produced from underground mining, and differences in the spatial resolution of the exposure variable.

Results from epidemiologic studies examining the relationship between diesel exhaust exposure during pregnancy and adverse birth outcomes are also relevant for comparison to the present study. In studies examining the composition of traffic-related PM_2.5_ in Los Angeles, increased exposure to diesel exhaust was shown to increase the odds of a PTB by 11% (95% CI = 7%, 15%) and a 5% (95% CI = 1%, 12%) increase in the odds of a birth being tLBW.^12,13^ Exposure to PM_2.5_ may mediate the association between proximity to active surface mining and birth outcomes found in this study; however, estimates of PM_2.5_ concentrations at residences around surface mines are currently not available. Polycyclic aromatic hydrocarbons (PAHs) and silica have been identified as specific components of particulate matter air pollution around Appalachian surface mining sites.^6,46,47^ Additionally, reduced ground and surface water quality has been characterized around surface mining sites in Central Appalachia^48,49^ and could be explored as a mediator of the relationship found here. Income disparities have decreased in the coalfield regions compared with elsewhere over the time period under study,^50,51^ and we have accounted for at least one proxy of economic status, payment method. Additionally, one unique aspect of this analysis is that we also compare to post-mining conditions, so, if decreasing employment opportunities were a contributing factor in the association seen, they would need to not only decrease during active mining compared with pre-mining time periods but would also need to increase again post-mining, which is not consistent with current economic analyses of the Appalachian region.^50–52^

Limitations of this analysis include potential confounding from unmeasured covariates that are temporally coincident with the land use change into active mining. We did not have information on mother’s alcohol use or nontobacco drug exposures during pregnancy, prenatal care, maternal body mass index (BMI), paternal demographics, marital status, or income. The current street-level analysis is also limited by the ability to geocode rural addresses with a high match rate. P.O. boxes accounted for approximately 40% of birth records; therefore, zipcode level, and not street-level exposure estimation, was the only exposure classification available for these records. This limitation reduces generalizability of our street-level findings and could have introduced bias into the analysis,^53^ while the low spatial resolution of the zipcode level analysis could introduce bias through exposure misclassification^54^; nevertheless, the consistent direction and strength of associations found in both analyses suggest bias, if present, may be minimal.

Relationships between surface mining activities and specific trimesters of exposure were not accounted for in this analysis as satellite imagery varies in quality and visibility by month, which limits accuracy of remote sensing analyses on a monthly time scale.^8,24^ Surface mining activities outside of Central Appalachia but neighboring the study area, other industries, and traffic-related sources of air pollution are not accounted for in this analysis. This study also assumed that mothers spent the totality of their pregnancy at the address or ZIP code listed on the birth records, as we did not have access to information on mobility during pregnancy, which can lead to exposure misclassification, as previous studies have noted, particularly for 1st and 2nd trimester exposures.^55–57^

Despite these limitations, this study adds to the knowledge of the potential health impacts of living near surface mining in Central Appalachia by analyzing individual birth records at two previously unstudied spatial scales, across a relatively long time period.

## Conclusions

This study found associations between amount of active surface mining within close proximity to residence during gestation and the odds of preterm birth, low birth weight, and term low birth weight. These results justify further research into the health burden associated with land use change in Central Appalachia.

## Conflicts of interest statement

The authors declare that they have no conflicts of interest with regard to the content of this report.

Supported by a grant from the National Institute of Environmental Health Sciences (R21ES028396).

## ACKNOWLEDGMENTS

We thank Chris Grubb and Suwei Wang for their assistance in data processing in R and Michael Marston for supplying surface mine delineation files based on Landsat data analysis.

## Supplementary Material


