# Supplementary material for: Maternal proximity to Central Appalachia surface mining and birth outcomes
Source: Environ Epidemiol. 2021 Jan 25;5(1):e128. doi: 10.1097/EE9.0000000000000128 (PMC7939414; doi:10.1097/EE9.0000000000000128)
Supplement: Supplementary file 1 [file ee9-5-e128-s001.docx]

##

## Supplemental Figures 1-3 and Supplemental Tables 1-9


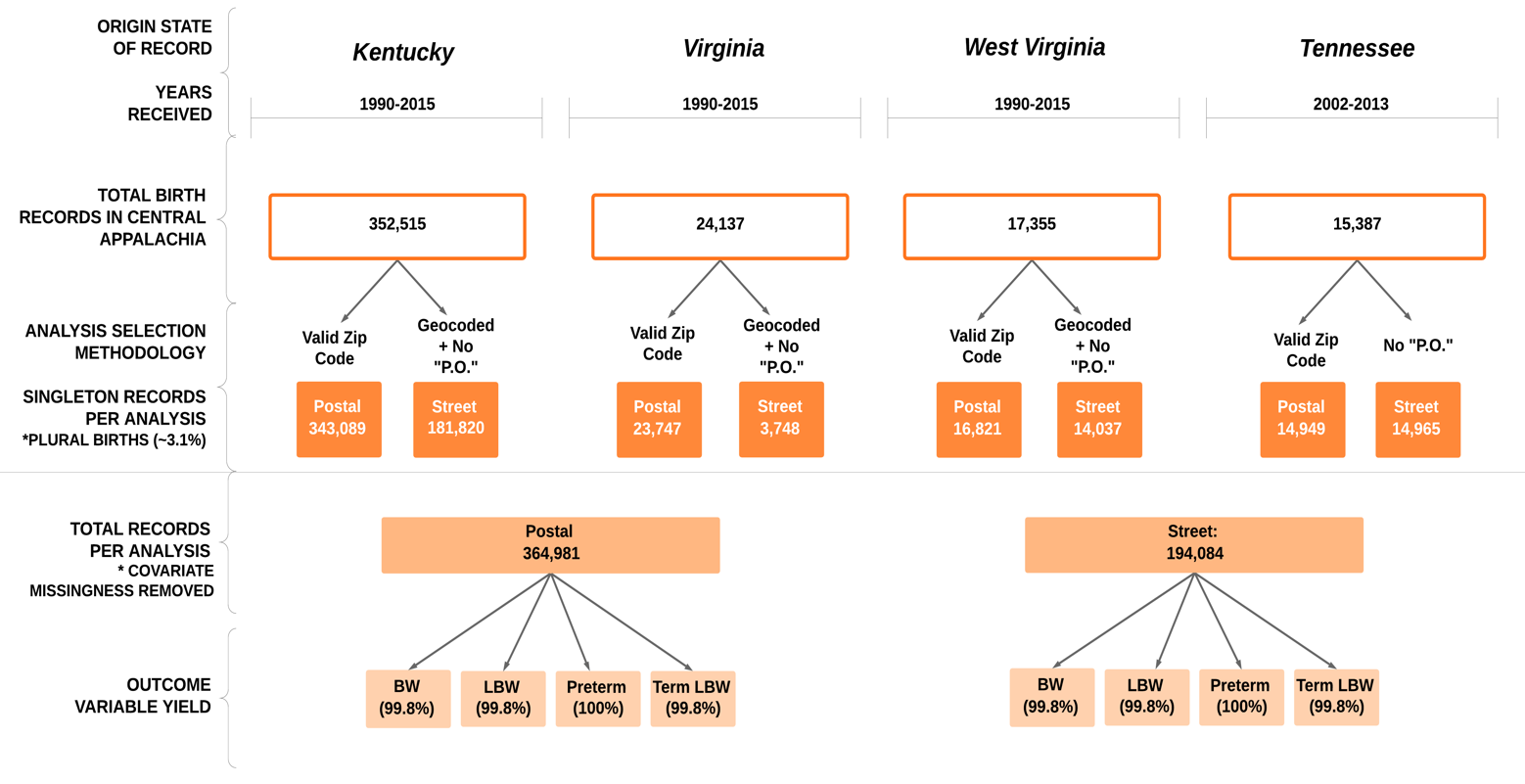


Supplemental Figure 1. Flow diagram of birth record data processing


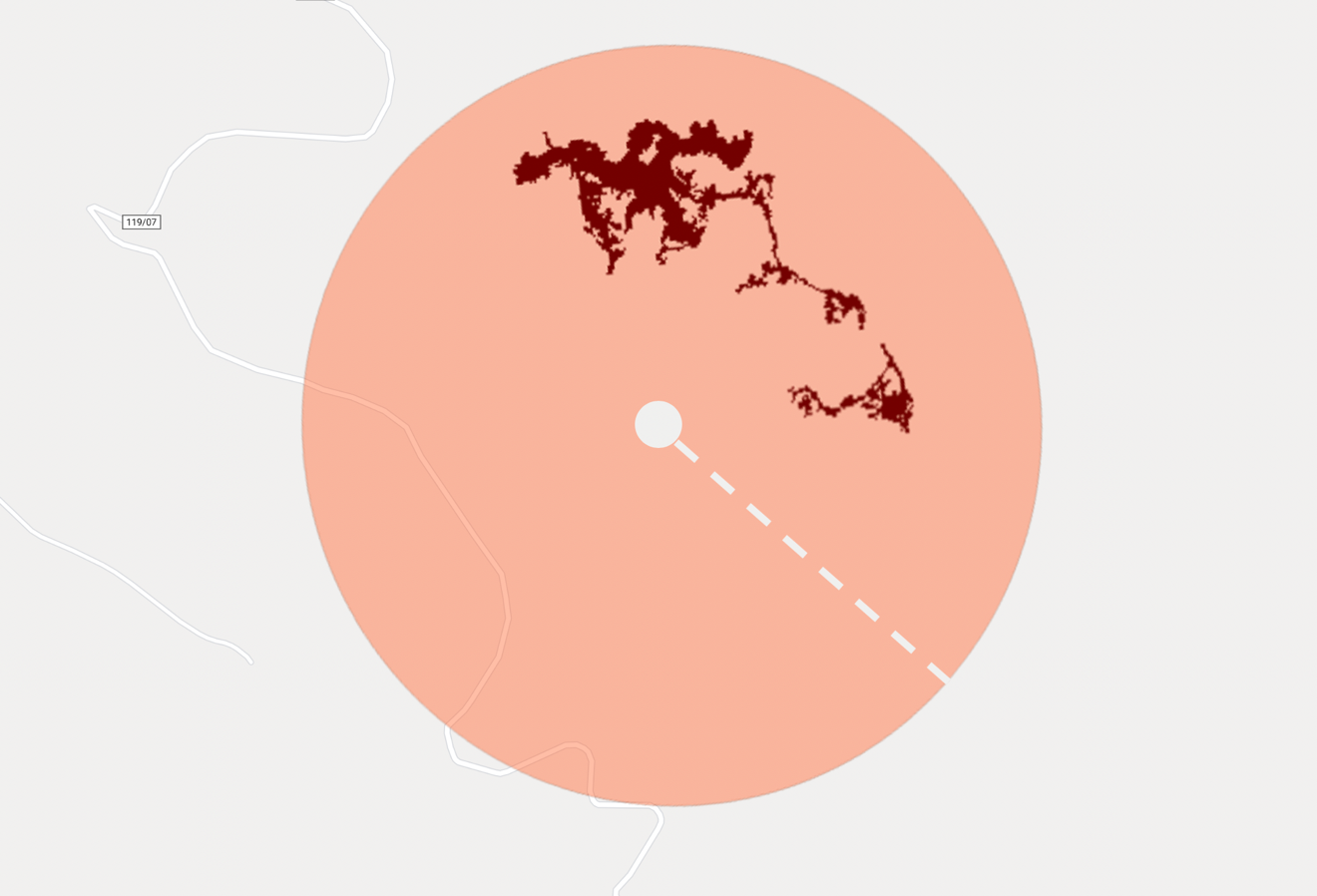


Supplemental Figure 2. A fictitious 5 km buffer of a mother’s address(orange) overlaid with 2010 active surface mining polygons(maroon) (address and surface mine location altered for privacy)

| 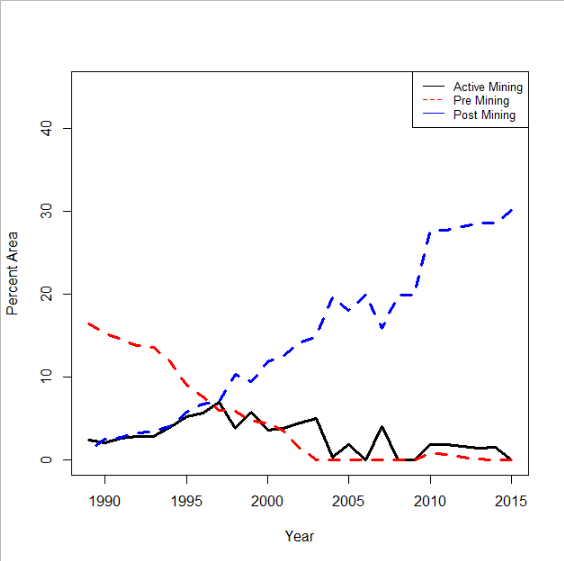 | 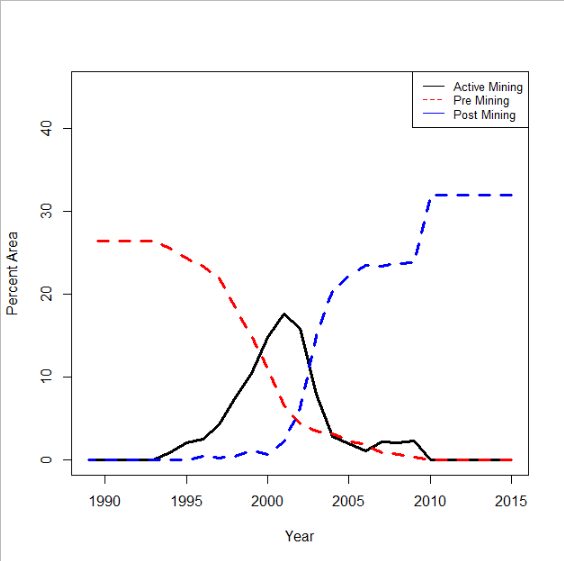 |
| --- | --- |
| 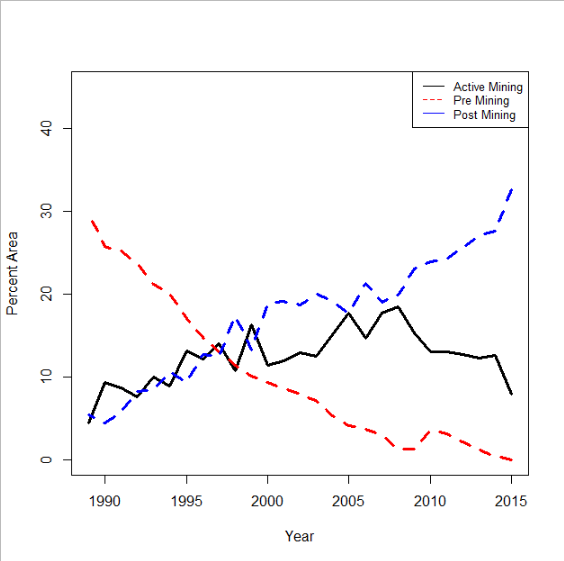 | 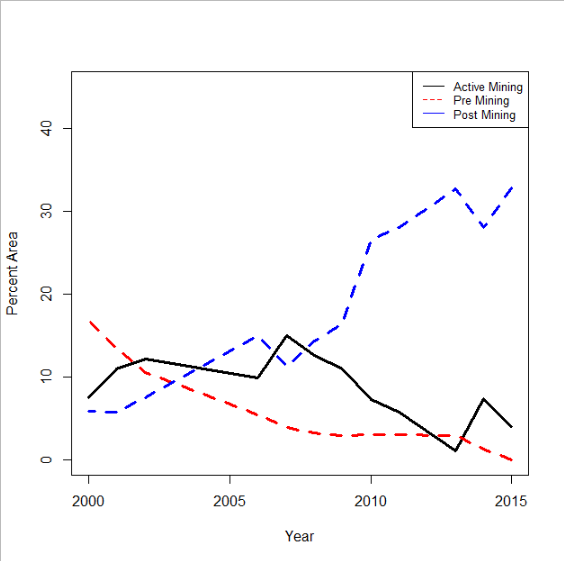 |
| Supplemental Figure 3. Progression of active mining in representative ZCTAs. The amount of pre and post-mining are determined from delineation of active mining in prior and subsequent years, respectively, between 1987 and 2015. Representative ZCTAs (41740, 41559, 41727, and 25193) with a relatively high amount of mining (greater than 30% mined) are shown. | |

Suppl Table 1. Change in birthweight (grams) and odds ratios (95% CIs) for percent of pre-mining area within 5 km of maternal residence

|  | Pre-Mining within 5 km of Maternal Residence  (per 1% increase) | |
| --- | --- | --- |
| **Outcome Variable** | **Grams or OR (95% CI)** | ***P*-value** |
| Birthweight | -0.61(-2.60, 1.38) | 0.549 |
| Preterm | 1.00(0.98,1.01) | 0.678 |
| Low Birth Weight | 1.01(1.00,1.03) | 0.114 |
| Term Low Birth Weight | 1.02(1.00,1.04) | 0.078 |
| Adjusted for mother's age, mother's race, mother's tobacco use during pregnancy, mother's education, parity, year of majority of gestation, State, and child's sex | | |

Supplemental Table 2. Change in birth weight and odds ratios (95% CIs) for associations between birth outcomes and amount of pre-mining within 5 km of maternal residence, additionally adjusted for Hispanic origin of mother and payment method

|  | Pre-Mining within 5 km of Maternal Residence  (per 1% increase) | |
| --- | --- | --- |
| **Outcome Variable** | **Grams or OR (95% CI)** | ***P*-value** |
| Birthweight | 1.60(-2.50, 5.70) | 0.444 |
| Preterm | 1.00(0.99,1.02) | 0.842 |
| Low Birth Weight | 1.02(0.99,1.05) | 0.296 |
| Term Low Birth Weight | 1.02(0.98,1.06) | 0.363 |
| Adjusted for mother's age, mother's race, mother's tobacco use during pregnancy, mother's education, parity, year of majority of gestation, State, Hispanic origin of mother, payment method, and child's sex | | |

Supplemental Table 3. Odds ratios (95% CIs) for associations between adverse birth outcomes and amount of active and post-mining within 5 km of maternal residence in adjusted logistic model

|  | Amount of Mining within 5 km of Maternal Residence | | | | |
| --- | --- | --- | --- | --- | --- |
|  | Active Mining (per 1% increase) | | Post-mining (per 1% increase) | |  |
| **Outcome Variable** | **OR (95% CI)** | ***P*-value** | **OR (95% CI)** | ***P*-value** |  |
| Preterm | 1.02(0.98,1.06) | 0.378 | 1.01(1.00,1.02) | 3.83e-02 |  |
| Low Birth Weight | 1.04(1.00,1.10) | 0.070 | 1.00(0.99,1.01) | 0.433 |  |
| Term Low Birth Weight | 1.07(1.00,1.16) | 0.060 | 1.00(0.99,1.02) | 0.793 |  |
| This regression was adjusted for mother's age, mother's race, mother's tobacco use during pregnancy, mother's education, parity, year of majority of gestation, State, and child's sex | | | | |  |

Supplemental Table 4. Odds ratios (95% CIs) for associations between adverse birth outcomes and amount of active mining within 5 km of maternal residence when logistic regression models were further adjusted for payment method and mother’s Hispanic origin

|  | Active Mining within 5 km of Maternal Residence  (per 1% increase) | |
| --- | --- | --- |
| **Outcome Variable** | **OR (95% CI)** | ***P*-value** |
| Preterm | 1.09(1.06,1.14) | 1.33e-06 |
| Low Birth Weight | 1.07(1.02,1.11) | 2.01e-03 |
| Term Low Birth Weight | 1.00(0.94,1.07) | 0.999 |
| This regression was adjusted for mother's age, mother's race, mother's tobacco use during pregnancy, mother's education, parity, year of majority of gestation, State, child's sex, payment method, and mother’s Hispanic origin | | |

Supplemental Table 5. Change in birthweight (grams) and odds ratios (95% CIs) for percent of pre-mining area within maternal residence ZCTA

|  | Pre-Mining within 5 km of Maternal Residence  (per 1% increase) | |
| --- | --- | --- |
| **Outcome Variable** | **Grams or OR (95% CI)** | ***P*-value** |
| Birthweight | 0.19(-0.64, 1.01) | 0.657 |
| Preterm | 1.00(0.99,1.00) | 0.703 |
| Low Birth Weight | 1.00(0.99,1.00) | 0.409 |
| Term Low Birth Weight | 1.00(0.99,1.01) | 0.821 |
| Adjusted for mother's age, mother's race, mother's tobacco use during pregnancy, mother's education, parity, year of majority of gestation, State, and child's sex | | |

Supplemental Table 6. Change in birth weight and odds ratios (95% CIs) for associations between birth outcomes and amount of pre-mining within maternal ZCTA, additionally adjusted for Hispanic origin of mother and payment method

|  | Pre-Mining within 5 km of Maternal Residence  (per 1% increase) | |
| --- | --- | --- |
| **Outcome Variable** | **Grams or OR (95% CI)** | ***P*-value** |
| Birthweight | -0.61(-2.82, 1.59) | 0.585 |
| Preterm | 1.02(1.00,1.03) | 0.039 |
| Low Birth Weight | 1.00(0.98,1.02) | 0.843 |
| Term Low Birth Weight | 1.00(0.98,1.03) | 0.690 |
| Adjusted for mother's age, mother's race, mother's tobacco use during pregnancy, mother's education, parity, year of majority of gestation, State, Hispanic origin of mother, payment method, and child's sex | | |

Supplemental Table 7. Odds ratios (95% CIs) for associations between adverse birth outcomes and amount of active and post-mining within maternal ZCTA boundary in adjusted logistic model

|  | Amount of Mining within Maternal ZCTA Boundary | | | |
| --- | --- | --- | --- | --- |
|  | Active Mining (per 1% increase) | | Post-mining (per 1% increase) | |
| **Outcome Variable** | **OR (95% CI)** | ***P*-value** | **OR (95% CI)** | ***P*-value** |
| Preterm | 1.02(1.00,1.05) | 0.095 | 1.01(1.01,1.02) | 6.31e-08 |
| Low Birth Weight | 1.04(1.01,1.07) | 9.92e-03 | 1.01(1.00,1.01) | 0.073 |
| Term Low Birth Weight | 1.06(1.02,1.10) | 4.21e-03 | 1.00(0.99, 1.01) | 0.830 |
| This regression was adjusted for mother's age, mother's race, mother's tobacco use during pregnancy, mother's education, parity, year of majority of gestation, State, and child's sex | | | | |

Supplemental Table 8. Odds ratios (95% CIs) for associations between adverse birth outcomes and amount of active within ZCTA boundary when logistic regression models were further adjusted for payment method and mother’s Hispanic origin

|  | Active Mining within ZCTA Boundary  (per 1% increase) | |
| --- | --- | --- |
| **Outcome Variable** | **OR (95% CI)** | ***P*-value** |
| Preterm | 1.08(1.06,1.10) | 2.34e-14 |
| Low Birth Weight | 1.06(1.04,1.09) | 2.65e-07 |
| Term Low Birth Weight | 1.03(0.99,1.07) | 0.144 |
| This regression was adjusted for mother's age, mother's race, mother's tobacco use during pregnancy, mother's education, parity, year of majority of gestation, child's sex, payment method, State, and mother’s Hispanic origin | | |

## Supplemental Table 9. Akaike information criterion for the models evaluated

|  | Street | | Postal | |
| --- | --- | --- | --- | --- |
|  | Main (n=194,084) | With Payment and Hispanic (n=95,464) | Main (n=364,981) | With Payment and Hispanic (n=171,726) |
| *Birthweight* | | | | |
| Pre-mining | 2995796 | 1475393 | 5632777 | 2652157 |
| Pre + Active | 2995772 | 1475373 | 5632724 | 2652085 |
| Pre+Active+Post | 2995768 | 1475365 | 5632662 | 2652042 |
| *Preterm Birth (PTB)* | | | | |
| Pre-mining | 119226 | 62318 | 220418 | 112543 |
| Pre + Active | 119216 | 62300 | 220394 | 112492 |
| Pre+Active+Post | 119212 | 62294 | 220369 | 112475 |
| *Low Birth Weight (LBW)* | | | | |
| Pre-mining | 95542 | 50801 | 180458 | 91990 |
| Pre + Active | 95537 | 50793 | 180447 | 91969 |
| Pre+Active+Post | 95539 | 50796 | 180447 | 91971 |
| *Term Low Birth Weight (tLBW)* | | | | |
| Pre-mining | 44202 | 24052 | 86067 | 44365 |
| Pre + Active | 44205 | 24055 | 86065 | 44366 |
| Pre+Active+Post | 44205 | 24057 | 86065 | 44368 |
